# The Body Size-Dependent Diet Composition of North American Sea Ducks in Winter

**DOI:** 10.1371/journal.pone.0065667

**Published:** 2013-06-05

**Authors:** Jean-François Ouellet, Cécile Vanpé, Magella Guillemette

**Affiliations:** 1 Département de Biologie, Chimie et Géographie, Université du Québec à Rimouski, Rimouski, Québec, Canada; 2 Québec-Océan, Université Laval, Québec, Canada; University of St Andrews, United Kingdom

## Abstract

Daily food requirements scale with body mass and activity in animals. While small species of birds have higher mass-specific field metabolic rates than larger species, larger species have higher absolute energy costs. Under energy balance, we thus expect the small species to have a higher energy value diet. Also the weight and time constraints for flighted and diurnal foragers should set a maximum to the amount of prey items taken in one meal and to the daily number of meals, respectively. Further, avoidance of competition causes the species to reduce the amount of shared prey in their diet. Some diet segregation is therefore to be expected between species. We tested these hypotheses and investigated the role of body mass in the diet composition of 12 sea duck species (*Somateria mollissima*, *Somateria spectabilis*, *Somateria fischeri*, *Polysticta stelleri*, *Bucephala clangula*, *Bucephala islandica*, *Bucephala albeola*, *Melanitta nigra*, *Melanitta perspicillata*, *Melanitta deglandi*, *Histrionicus histrionicus* and *Clangula hyemalis*) wintering in North America. This study was based on a literature survey with special emphasis given to the diet data from the former US Bureau of Biological Survey. The data supported our hypothesis that the energy value of winter diet of sea ducks scales negatively with body mass. Diet diversity also scaled negatively with body mass. Our results suggest the existence of a minimum for the energy value of avian diets.

## Introduction

An animal must balance energy intake with energy expenditures to maintain body condition and survive. Because field metabolic rate scales allometrically with body mass [1], [2], [3], [4], small animals have greater energy and nutrient requirements relative to their body mass than large species. As a result, small animals have lower capacities to withstand prolonged periods of food scarcity [5]. Therefore, it can be expected that the body mass of an animal influences its food choice and foraging strategy.

For a foraging animal, obtaining food can be broken down into a series of activities such as searching for a prey patch, capturing and handling that prey, and digestion while defending against competitors and avoiding predators. Each step in this array of processes takes time and energy and can potentially contribute to regulate the rate of assimilation in a consumer. A vast body of literature emphasizes pre- or post-ingestion processes [6], [7], [8], [9], [10]. Foraging theory predicts [11] that a forager should select the food items that maximize long term average rate of energy intake. A foraging animal, however, must also reconcile a variety of constraints and make decisions as to the satisfying trade-offs. For example, prey preference changes according to ambient temperature, body condition, prey availability, competition pressure and predation risk [12], [13], [14], [15], [16], [17], [18]. Also, this set of constraints is exacerbated when the daily time available for foraging and processing prey is limited, for instance during winter in temperate and boreal regions [8], [19], [20], [21].

Two strategies that allow a forager to achieve energy intake maximization are to rely on food quality and maximize net energy intake or on food quantity and maximize gross energy intake [6], [22], [23], [24]. On one hand, successful quality strategists and/or energy maximizers avoid filling their gut with average quality food and thereby wasting better opportunities to feed on highly energetic prey. This strategy is associated with a high energy gain for each item ingested, but imposes longer search time and lower encounter rate. Quality strategists are often risk prone and deal with regulating factors such as low food density or prey's ability to escape or hide. Because of the unpredictability associated with high quality prey, quality strategists may be forced to rely on a wider diversity of prey than quantity strategists [25], [26]. On the other hand, quantity strategists must rely on food that ensures a high encounter rate and minimal search time with little regard of energy value. This strategy allows securing a large amount of food items within a short period of time. However, if the energy value of food is low and ingestion rate exceeds the rate of the digestive processes, the forager ends up gorged with food and must structure its foraging activity in several ingestion bouts with intervening rest interludes [6], [27], [28]. Obviously, either strategy may severely challenge the energy budget of a time-constrained forager. When offered a variety of prey, a forager must adopt the strategy which offers the best probability to meet its requirements before it runs short of time. According to the energy budget rule and risk-sensitivity principle, foragers expecting a positive energy balance in the short term are more likely to select prey in the risk-averse fashion (i.e. favor probability of success more than reward) whereas the risk-prone behavior is adopted by foragers in poor condition or expecting a negative energy balance [6], [13], [29], [30].

Wintering sea ducks offer an appropriate system to investigate the role of the body mass in diet composition due to their life history strategies and marine habitats. Sea ducks belong to a monophyletic tribe and are spread over a wide spectrum of body mass [31], [32], [33]. Their coastal habitats are colonized by a variety of potential prey that vary in energy values, numeric densities and vulnerability to predation. For sea ducks living in open water habitats, the daily energy budget is challenged by the high costs of thermoregulation [34], [35], [36], diving [37] and flight [38] as well as the limited energy income due to the low quality of benthic food resources [6], [39], [40], [41]. In addition, the daily foraging schedule of these diurnal predators is possibly limited by short winter photoperiods, tidal oscillations and adverse weather [8], [19], [20], [21], [42], [43]. Their small wings and flight musculature relative to their body mass impose on them a laborious take off and any extra weight (mass times gravitational acceleration), like that of a large amount of prey stored in the gut, may impair their take off capability [44], [45], [46]. For these reasons, the adoption of a winter diet that maximizes the probability to achieve energy balance is likely a process governed by strict rules with little left to chance. With respect to scaling principles, we expect winter conditions to challenge the daily energy budget of small species more than that of large ones. Small species are therefore expected to show a stronger propensity to forage in the risk-prone fashion and rely on a wider diversity of prey and on prey with higher quality than large ones. Also, in accordance with the competitive exclusion principle [47], these sympatric sea duck species, in order to coexist and if their resources are limited, should avoid niche overlap, either by feeding on different prey, and/or by feeding on the same prey but of different size, and/or by feeding on the same prey at different time of the day or at different depths.

In this paper, we investigated the diet of sea duck species wintering in North American coastal environments. We specifically tested the hypothesis stating that the energy content of the winter diet of sea ducks scales negatively with their body mass. Such a relationship was reported by Goudie and Ankney [48] on a local scale for four sea duck species. Here, we tested our hypothesis at the scale of North America with 12 species. Also, these authors observed a negative relationship between diet diversity and body mass and we hypothesized that the winter diet of larger species is focused on a narrower variety of prey than smaller species.

## Methods

### Ethics Statement

This study is restricted to data analyses and, therefore, excludes any animal handling or invasive experiments. The study thus adheres to the “Guidelines for the Use of Animals in Research”, and to the legal requirements of the country in which the work has been carried out.

### Diet composition

We used a data set from the former Bureau of Biological Survey of the United States Department of Agriculture (hereafter USBS) spanning several decades (1885 to 1965). The USBS brought together a vast collection of waterfowl specimens collected across North America by hunters and ornithologists. The digestive tract of the specimens was dissected by the Food Habits laboratory staff and the content of gizzard and esophagus was described quantitatively, tabulated and archived. We were granted access to the raw data set of these gut content examinations. No individual duck was collected specifically for this study.

For each individual bird, the following information was recorded: identification number, species, date and collection location, name of investigator, content of gut relative to the total gut capacity, relative volume of each prey species. We focused our investigation on sea duck species wintering in North America and feeding on marine invertebrates, therefore excluding ichthyophagous *Mergus* species. Our analysis includes only diet composition data obtained through direct gut content examination on specimens collected in coastal waters of North America between September and April. Unfortunately, the information on sex and age of the individuals was generally lacking. This precluded any possibility to take sexual or age-mediated dimorphism into account.

This data set gave emphasis to sea duck wintering grounds that were known at the time of collection, thereby excluding some important wintering grounds discovered recently like those of the spectacled eiders in the Bering Sea [49] and long-tailed ducks in Nantucket Shoals [50]. We supplemented the data set with a literature review for the species that had less than approximately 60 individuals in the USBS data set. In such cases, in order to avoid giving too much weight to a single geographic origin of specimens, we combined one source from the East and one from the West coast of North America when possible. Sources reporting diet composition with frequency of occurrence data were discarded.

Our study of diet composition includes a total of 912 individuals distributed in 12 species as shown in Table 1.

**Table 1:** Sea duck species included in the diet composition data set and their respective number of gut contents and collection sites.

Common name	Latin name	Nb of gut contents	Nb of collection sites
Common eider	*Somateria mollissima*	100	11
King eider	*Somateria spectabilis*	32	5
Spectacled eider	*Somateria fischeri*	14	2
Steller's eider	*Polysticta stelleri*	8	3
Common goldeneye	*Bucephala clangula*	119	7
Barrow's goldeneye	*Bucephala islandica*	70	11
Bufflehead	*Bucephala albeola*	59	22
Black scoter	*Melanitta nigra*	59	17
Surf scoter	*Melanitta perspicillata*	99	26
White-winged scoter	*Melanitta deglandi*	185	nd
Harlequin duck	*Histrionicus histrionicus*	94	10
Long-tailed duck	*Clangula hyemalis*	73	9

We focused our analysis at the level of the taxonomic class of prey. We believe it relevant to mention that malacostraca refers to the class grouping amphipods, decapods, and isopods. For computational convenience, categories of prey “unidentified”, “other animals”, “grit” and “plant remains” were removed from the data set. For each sea duck species, the relative contribution of each prey taxa to the total amount of prey was averaged over every individuals of that species in the USBS data set. When additional data sources were necessary, a weighted mean was calculated over each intra-source average values. The use of weighted means equalized the weight of each individual sampled. We used the number of prey taxa in the diet and prey dominance (relative contribution of the most represented taxon in the diet) as an index of diet diversity. These variables were averaged according to the same procedure as relative contribution for prey. We tested the correlation between these variables and body mass. Note that our approach does not account for sexual dimorphism, local variations in diet or prey switches that may occur over the course of a winter; but it has the advantage of damping out the effect of local and annual fluctuations of prey abundance on prey selection by sea ducks.

### Body mass

Body mass data were taken from published figures and averaged over multiple sources. Our study includes only data from sources reporting body mass during winter. Mean body mass was calculated according to the following procedure: one mean value for each sex was calculated over each source reporting sexes separately; these sex-specific mean values were averaged into a mean value for the species (both sexes combined). This value was averaged together with mean values from sources reporting sexes pooled in order to provide a mean value for the species. We used a weighted mean in all instances except when averaging sexes together. This procedure tended to equalize the weight of each sex and to reduce the potential for bias caused by annual or regional variations in body mass. Standard deviations were not weighted since they were often lacking in published figures. When necessary, values were log-transformed to improve linearity in correlation analyses.

### Energy value of diets

Data on energy content of prey taxa were also taken from the literature (See Results). All values were expressed in kilojoules per gram of wet weight including the exoskeleton. When converting from a dry weight to a wet weight basis was necessary, we used the moisture content value provided along with the energy content. We calculated the energy value, on a per gram basis, of the gut content for every specimen in the USBS data base. For each species we calculated a weighted mean over each source of data. Standard deviations were not weighted since they were often lacking in published figures. We tested the correlation between log-transformed values of energy value of the diets and body mass. We also tested this correlation after segregating the specimens according to their Eastern or Western Coast origin.

### Relative intake

In order to investigate the potential consequences of diet composition and scaling on daily food intake, we estimated the relative daily prey intake needed by sea ducks to match their requirements. For that purpose, we built up hypothetical scenarios where fictive species fed on a unique taxon. Our sample consisted of 12 fictive duck species under energy balance and weighing from 400 to 2100 g. Prey taxa were bivalves and malacostraca which were the most widely represented prey in the diets (see Results). We considered the daily energy requirements to be equal to their daily energy expenditures (DEE) divided by an assimilation efficiency coefficient. Daily energy expenditures were calculated according to the procedure of Miller and Eadie [51] (model for all ducks). Energy content of prey was mean ± SD (see Results). We used an assimilation efficiency coefficient of 70% for bivalves as measured by Richman and Lovvorn [16] on common eiders, and 60% for malacostraca as calculated from Jorde and Owen [52] on black ducks (*Anas rubripes*). The latter is possibly a conservative value when applied to sea ducks. Sea ducks, being strict carnivorous species in winter are probably not less efficient than the black duck [53] in digesting animal matter.

### Phylogenetic constraint

Our sample consisted of 12 species but only six genera. *Histrionicus*, *Clangula* and *Polysticta* are monospecific genera but *Bucephala*, *Melanitta* and *Somateria* contain three species each. This phylogenetic background gives way to a potential phylogeny mediated bias since congener species are not independent from one another [54], but see [55]. In order to factor out the phylogenetic background, we conducted our analysis at the species and at the genus levels in parallel. We assumed the six genera to be independent taxonomic entities. This assumption is supported by the phylogenies of sea ducks published by Livezey [31] and Donne-Goussé et al. [33].

## Results

### Body mass

At the inter specific level, body mass ranged from 492±63 g for the bufflehead to 1922±170 g for the common eider (mean ± SD = 1145±471 g, median = 1056 g; Table 2). Species above the median were the eiders belonging to the genus *Somateria* and scoters. At the inter genus level, the body masses ranged from 643±58 to 1767±152 g for *Histrionicus* and *Somateria*, respectively. Inter genus mean and median were 1009±434 and 797 g, respectively. The order of increasing body mass is consistent across the two taxonomic levels with the exception that Steller's eider, which belongs to the monospecific genus *Polysticta*, switches from well below median at the inter specific level to above median at the inter genus level.

**Table 2:** Mean body mass of the 12 studied sea duck species and their respective six genera, according to a literature review.

	Mean body mass (g)	n	Sources
**SPECIES**			
Bufflehead	492±63	99	[76], [77], [78]
Harlequin duck	643±58	715	[79], [80], [81], a
Long-tailed duck	761±64	49	[78], [82], a
Steller's eider	809	285	[80]
Common goldeneye	882±213	39	[56], [78], [82], b
Barrow's goldeneye	978±195	102	[56], [81], [82], b
Black scoter	1133±94	105	[80], a
Surf scoter	1153±35	103	[78], [83]
White-winged scoter	1590±236	77	[78], [80]
Spectacled eider	1619±98	38	[84]
King eider	1759±120	55	[80], c
Common eider	1922±170	65	[48], a
**GENERA**			
Histrionicus	643	1	
Clangula	761	1	
Bucephala	784±258	3	
Polysticta	809	1	
Melanitta	1292±258	3	
Somateria	1767±152	3	

Note: Species and genera are presented in increasing order of mass. n represents the total number of individuals included in the mean (see Methods for details on computations). Data are means ± SD.

a: P.L. Flint, unpublished data;

b: J.-F. Ouellet, unpublished data;

c: F. Merkel, unpublished data.

### Diet composition and diversity

**Figure 1:**
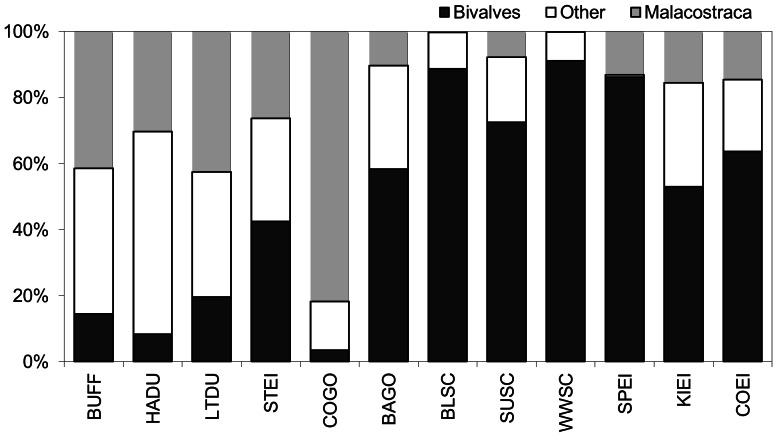
Contribution of bivalves, malacostraca and other prey to sea duck winter diet. The 12 studied sea duck species are presented in order of increasing body mass. Species codes are same as Table 3.

The most widely represented prey classes in the diet of sea ducks were bivalves (mean ± SD = 50.1±32.2%), malacostraca (23.7±23.2%) and gastropods (13.7±10.0%) (Table 3). These taxa were present in the diet of almost every species, although in varying amount. Figure 1 suggested the existence of an increasing contribution of bivalves with increasing body mass (arc-sine transformed values, r^2^ = 0.482; p = 0.012) and the opposite trend for malacostraca. The same trend was observed at the genus level (bivalves: r^2^ = 0.669; p = 0.047) with arc-sine transformed values. The common goldeneye exhibited the largest contribution of malacostraca (81.8±26.0%) and the smallest contribution of bivalves (3.5±14.4%) (Fig. 1). Finally, gastropods tended to be more represented in the diet of smaller species or genera than in larger ones (Table 3).

**Table 3:** Winter diet composition of the 12 studied sea duck species.

		BUFF	HADU	LTDU	STEI	COGO	BAGO	BLSC	SUSC	WWSC	SPEI	KIEI	COEI	
**Nb of individuals**		59	94	73	8	119	70	59	99	185	14	32	100	
**Nb of sites**		22	10	>9	3	7	11	17	26	n.a.	2	5	11	
**Moll.**	**biva.**	14.5±26.4	8.3±6.2	19.5±9.5	42.5±43.5	3.5±14.4	58.3±35.6	88.7±25.7	72.4±41.2	91.1±3.3	86.2±68.2	53.0±39.1	63.6±43.9	
	**gast.**	22.6±31.4	27.5±12.4	20.1±15.2	31.1±32.8	1.7±6.3	13.5±8.2	5.0±17.3	9.2±26.9	5.9±4.1	0.1±0.7	13.2±19.9	14.1±30.1	
	**poly.**	0.9±5.4	12.1±8.2	0.0±0.0	0.0±0.0	0.0±0.0	0.1±0.1	0.1±0.4	0.0±0.0	0.0±0.0	0.0±0.0	1.5±7.3	0.1±0.7	
	**others**	0.0±0.0	0.2±0.4	0.5±0.6	0.0±0.0	0.5±4.2	1.5±2.2	0.9±5.1	0.7±4.7	0.0±0.0	0.0±0.0	0.4±2	0.0±0.0	
**Crus.**	**mala.**	41.5±39.2	30.3±21.1	42.6±18.7	26.3±40.5	81.8±26	10.3±8.0	0.3±1.7	7.8±23.7	0.2±1.1	13.1±64.9	15.5±29.6	14.6±28.9	
	**cirr.**	0.6±2.3	1.2±1.9	0.4±0.7	0.1±0.4	0.3±1.5	6.8±6.8	4.2±17.5	2.7±13.0	0.0±0.0	0.1±0.4	0.8±3.1	1.1±5.4	
	**others**	9.6±16.1	1.5±2.9	9.0±8.6	0.0±0.0	8.8±17.1	5.9±39.8	0.0±0.1	0.3±1.7	0.0±0.0	0.0±0.0	0.1±0.4	0.1±0.5	
**Echi.**	**aste.**	0.0±0.0	0.9±1.3	0.0±0.1	0.0±0.0	0.0±0.0	0.1±0.1	0.5±3.9	0.0±0.0	0.0±0.0	0.1±0.4	3.5±14.9	2.1±10.9	
	**ophi.**	3.4±16.4	0.2±0.9	0.0±0.0	0.0±0.0	0.0±0.0	0.0±0.0	0.0±0.0	0.0±0.0	0.0±0.0	0.0±0.0	0.0±0.0	0.2±2.2	
	**echi.**	0.0±0.0	0.8±1.1	0.0±0.0	0.0±0.0	0.0±0.0	0.1±0.1	0.1±0.4	1.7±12.3	2.8±2.2	0.3±1.5	6.2±19.0	2.9±14.4	
	**holo.**	0.0±0.0	0.0±0.0	0.0±0.0	0.0±0.0	0.0±0.0	0.0±0.0	0.0±0.0	0.0±0.0	0.0±0.0	0.0±0.0	0.0±0.0	0.0±0.3	
**Anne.**		3.9±14.5	0.9±3.6	0.7±1.6	0.0±0.0	0.6±5.9	0.6±1.3	0.0±0.0	1.1±10.1	0.0±0.3	0.0±0.0	0.2±1.0	1.0±8.5	
**Fish**		3.1±14.5	16.0±15.6	7.0±5.7	0.0±0.0	2.7±11.6	2.8±4.1	0.3±2.3	4.0±17.7	0.0±0.0	0.1±0.4	5.5±18.0	0.2±1.8	
**Sources**			a	[48], [71], a	[48], [78], a	a	a	[82], [85], a	a	a	[78], [86]	[84], a	a	a

Note: Values are mean ± SD percent of either weight or volume. Species are presented in order of increasing body mass. BUFF: bufflehead; HADU: harlequin duck; LTDU: long-tailed duck; STEI: Steller's eider; COGO: common goldeneye; BAGO: Barrow's goldeneye; BLSC: black scoter; SUSC: surf scoter; WWSC: white-winger scoter; SPEI: spectacled eider; KIEI: king eider; COEI: common eider; Moll.: mollusks; Crus.: crustaceans; Echi.: echinoderms; Anne.: annelids; biva.: bivalves; gast.: gastropods; poly.: polyplacophora; mala.: malacostraca; cirr.: cirripeds; aste.: asteroids; ophi.: ophiuroids; echi.: echinoids; holo.: holothuroids.

a: USBS.

**Figure 2:**
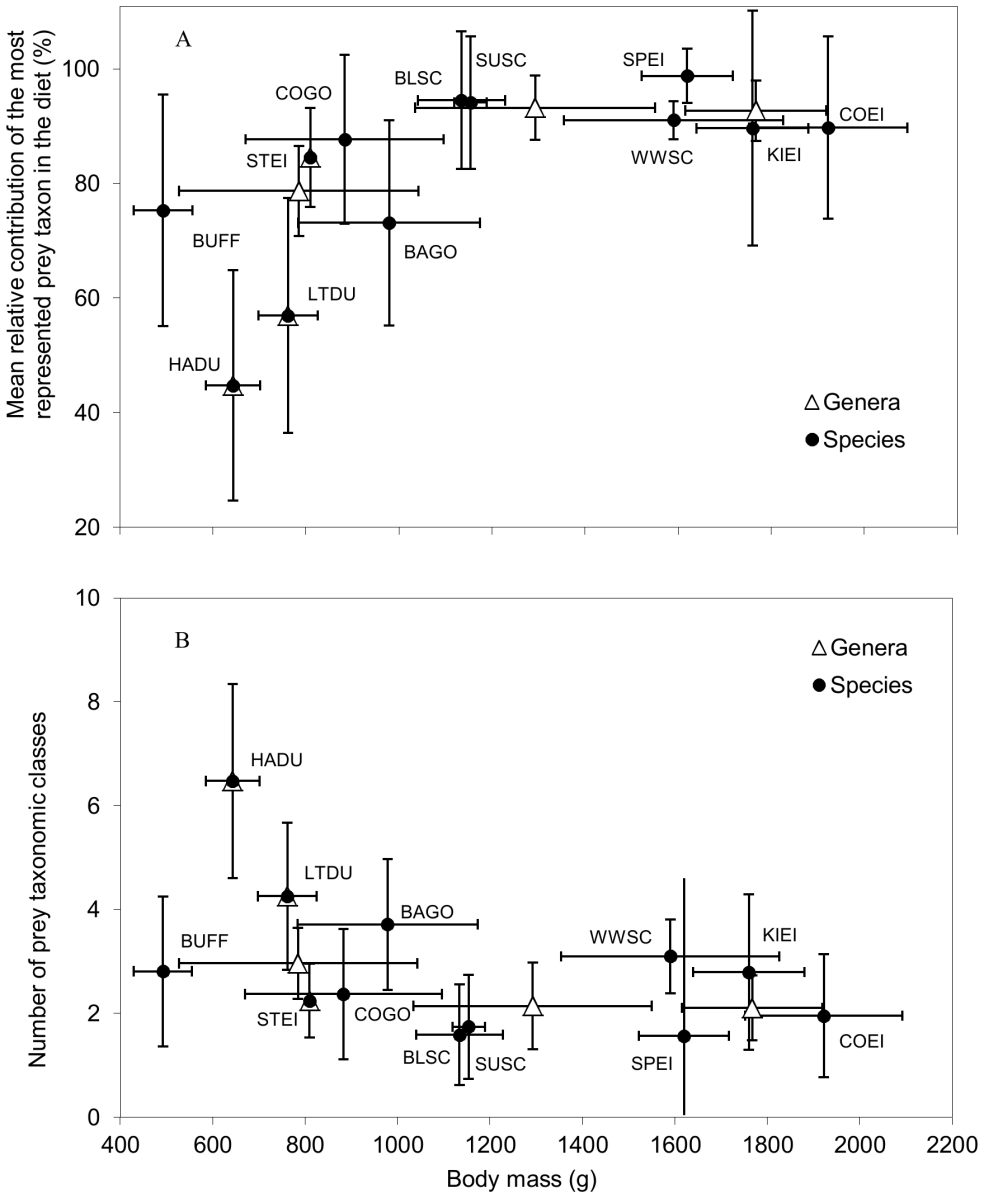
Relationship between two diet diversity indicators and body mass in sea duck winter diet. The two diet diversity indicators are: (A) mean relative contribution of the most represented prey taxon in the diet and (B) the number of prey taxa in the winter diet of invertebrate eating sea ducks in North America. Species codes are same as Table 3. The sequence of genera in order of increasing body mass is *Histrionicus*, *Clangula*, *Bucephala*, *Polysticta*, *Melanitta* and *Somateria*.

The prey dominance, as an index of diet diversity, scaled positively with body mass (Fig. 2A) (arc-sine transformed values, r^2^ = 0.412; p = 0.024 and r^2^ = 0.563; p = 0.086 for species and genus levels, respectively). The number of prey taxa in the diet seemed poorly related to body mass at either taxonomic level (Fig. 2B) (log-transformed values, r^2^ = −0.242; p = 0.104 and r^2^ = −0.554; p = 0.090 at the species and genus levels, respectively).

### Energy value of prey

Mean energy value of invertebrate prey (wet weights) ranged from 1.260±0.712 to 3.503±1.123 kJ.g^−1^ for echinoids and malacostraca, respectively (Table 4). Bivalves and gastropods were at the lower end of the range of values with 1.47±0.60 and 1.83±1.18 kJ.g^−1^. Holothuroids were nearly similar to malacostraca. Fish were well above the range of value of invertebrates with 5.53±1.53 kJ.g^−1^. Energy value of these organisms scaled negatively with their locomotor capacities, with holothuroids as the exception. Lowest values were found in sessile or slow moving and hard shelled organisms like echinoids, bivalves or gastropods. In contrast, highly mobile organisms like malacostraca or fish had the highest energy values (Table 4).

**Table 4:** Mean energy value (wet weights) of the most common prey taxa found in the winter diet of the 12 studied sea duck species, according to a literature review.

Phylum	Class	Mean energy value (kJ.g^−1^)	Range	Sources
Mollusks	bivalves	1.47±0.60	0.39–2.65	[40], [41], [52], [71], [87], [88], [89], [90], a
	gastropods	1.83±1.18	0.74–3.08	[41], [2], a
Echinoderms	echinoids	1.26±0.71	0.58–2.00	[6], [41], [87]
	ophiuroids	2.05±0.37	1.66–2.67	[39], [41], [89]
	asteroids	2.69±0.65	2.08–3.40	[41], [87]
	holothuroids	3.40		[41]
Crustaceans	cirripeds	2.05±0.05	2.01–2.08	[71]
	malacostraca	3.50±1.12	1.46–5.40	[6], [40], [41], [52], [71], [87]
Annelids	polychaetes	3.03±0.89	2.03–4.07	[71], [87], [89]
Chordates	fish and fish eggs	5.53±1.53	1.70–10.60	[41], [71]

Note: Data are means ± SD.

a: M. Guillemette, unpublished data.

### Scaling of the energy value of diets

**Figure 3:**
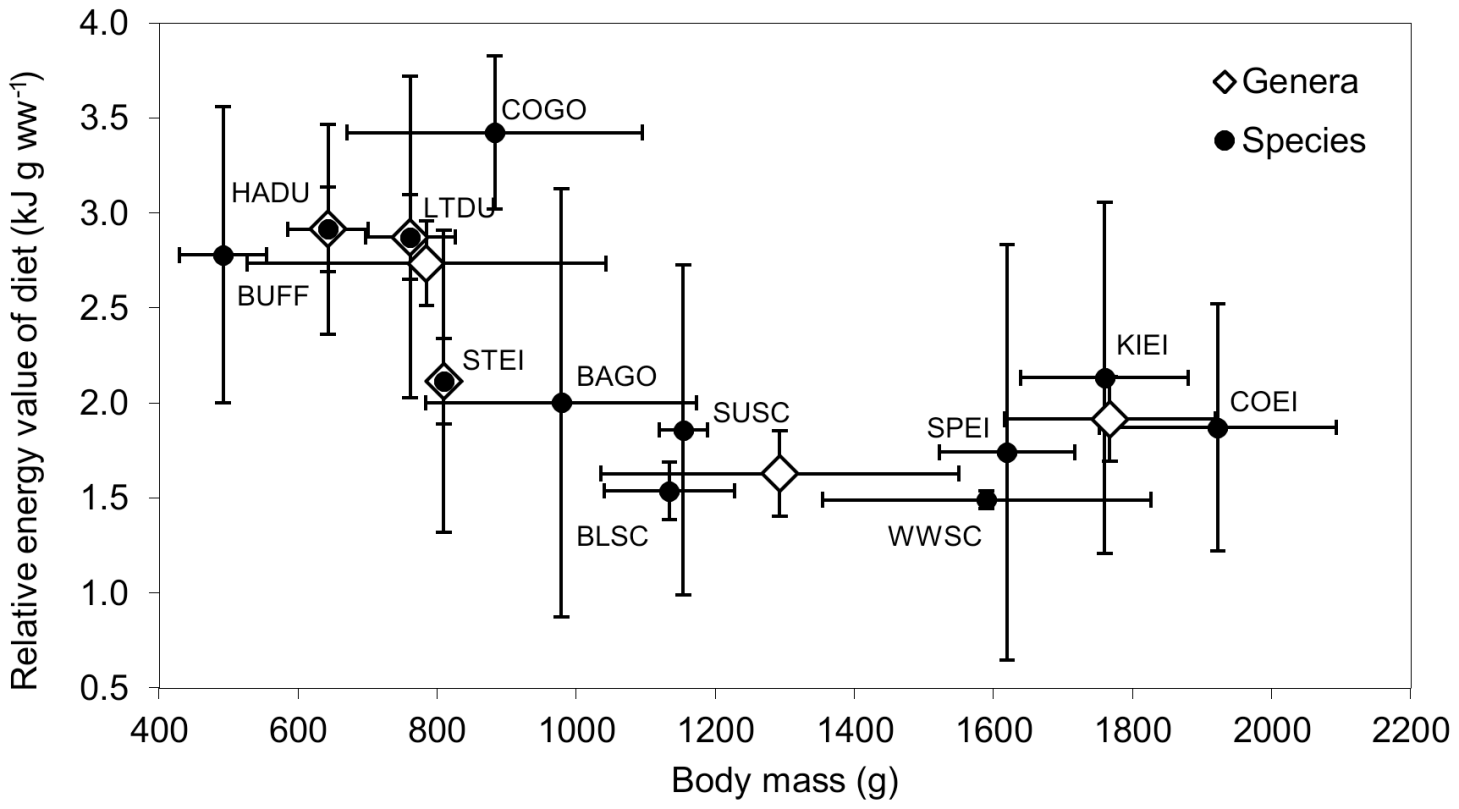
Energy value of the winter diet of sea duck species and their six respective genera. Species codes are same as Table 3. The sequence of genera in order of increasing body mass is *Histrionicus*, *Clangula*, *Bucephala*, *Polysticta*, *Melanitta* and *Somateria*. Energy values (in kJ.g.ww^−1^) were estimated according to a review of the literature. Error bars are for standard deviation.

The energy value of diet was negatively correlated with body mass and the results are consistent across taxonomic levels (EC = 1.644 M_b_^−0.433^, r^2^ = 0.479, p = 0.013; and EC = 1.888 M_b_^−0.512^, r^2^ = 0.661, p = 0.049 for the species and genus levels, respectively, where EC is energy content in kJ.g^−1^ and M_b_ is body mass in g) (Fig. 3). The relationship was still apparent after breaking down the data set in Atlantic (r^2^ = 0.621, p = 0.007, n = 10 species) and Pacific (r^2^ = 0.283, p = 0.075, n = 12 species) coasts (results not shown).

### Relative intake

**Figure 4:**
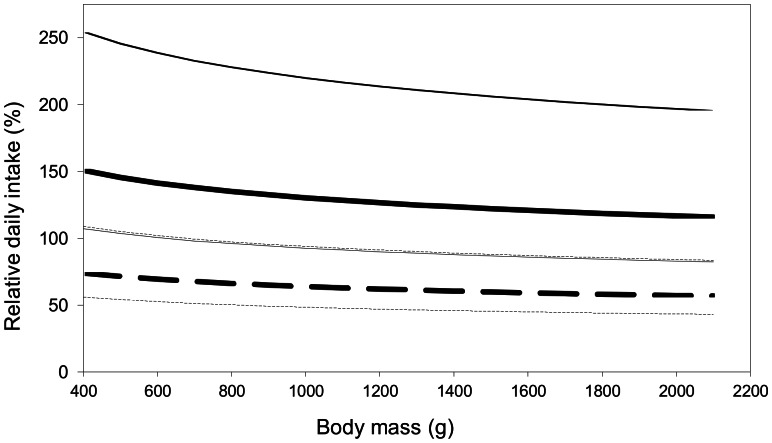
Daily prey intake relative to body mass for the 12 sea duck species. The daily intake was computed from daily energy expenditures derived from Miller and Eadie's [51] model for all ducks species. The analysis was based on the hypothesis that the species feed exclusively on either bivalves (solid lines) or malacostraca (dashed lines). Relationships were calculated with mean (bold lines) ± SD (thin lines) energy values (same as in Table 4).

If every species fed on the same unique prey taxon, small species would require a larger intake relative to their body mass than would large species, regardless of prey taxon (Fig. 4). A diet based solely on bivalves requires a larger intake than one based on malacostraca, regardless of the predator's body mass. For example, Figure 4 shows that an average bufflehead (body mass: 492 g) feeding exclusively on bivalves would require daily an intake exceeding its body mass (146%). If it fed on malacostraca only, its daily intake would represent 72% of its body mass. This discrepancy holds for larger species as well, but with smaller relative intake. This contrast between both exclusive diets is sharp despite that we possibly used a conservative assimilation efficiency coefficient for malacostraca.

## Discussion

In support to our first hypothesis stating that the energy content of the winter diet of sea ducks scales negatively with their body mass, our analyses revealed the existence of a relationship between the body mass and the energy value of the winter diet of sea ducks. This relationship was consistent across two taxonomic levels and both the Atlantic and Pacific coasts despite large differences in their benthic communities. This result is consistent with Goudie and Ankney's [48] findings with a smaller array of species over a smaller spatial scale. Although the aggregation of species into genera is phylogenetically appropriate, it reduced the variability by half. For example, the *Bucephala* genus contains the smallest species together with two medium sized species. Therefore, an average *Bucephala* is probably a weak representation of those species. Our dataset did not discriminate the highly energetic fish eggs from fish flesh. Fish were more widely represented in the diet of the harlequin duck and it is probable that a significant portion of these items were actually eggs [56], [57], [58]. The energy content of the harlequin's diet was probably underestimated, making the relationship seem even less steep than it is. The wide variability associated with some of the energy values of prey shown in Table 4 was a matter of concern. We conducted a sensitivity analysis in order to verify the impact of this variability on the validity of our results. This analysis was conducted at the species level and included bivalves and malacostraca which were the two most important prey (Table 4) and whose energy values were at both extremes of the gradient. The relationship between the energy value of the diet and body mass was still apparent (EC = 0.796M_b_^−0.146^; r^2^ = 0.437; p = 0.019) even after the relationship had been artificially flattened by adding one SD unit to the mean energy value of bivalves and subtracting one SD unit from the energy value of malacostraca.

If we examine this relationship from the upper end of the mass spectrum, we see an increasing quality of the diet as the consumers' body mass decreases (Fig. 3). This is consistent with scaling principles stating that small species have higher mass-specific food requirements than large species. We conclude that smaller species adhere to the prey quality strategy under a physiological constraint possibly exacerbated by a tight foraging schedule. However, this explanation is not satisfactory for someone looking at the relationship from the lower end of the mass spectrum. There is probably no physiological constraint that prompts the large sea ducks to take the lowest energy value food (Fig. 3).

In larger sea ducks, prey preference is clearly driven by another currency than energy value. A possible alternative currency is maximization of intake rate. Most prey fed upon by larger species, like bivalves and echinoderms, are sessile or nearly so and can reach large numerical densities in suitable habitats. This probably reduces search and capture time to a minimum compared with mobile prey. This allows a forager to secure a large amount of prey within a short period of time, and to proceed later with digestion should foraging conditions deteriorate. This is a valuable advantage in temporally structured ecosystems like northern coastal environments. Conversely, these prey often have anti-predator armor such as being protected with a hard mineral exoskeleton, which is swallowed along with the flesh by sea ducks, and have a lower than average energy value. The mechanical crushing of the hard shell by the gizzard of a duck is a lengthy process. Since the digestion rate is much lower than the ingestion rate, the forager ends up gorged with prey items and must structure its foraging activity in a cycle where ingestion bouts alternate with digestion interludes. An extreme example of such a time structure of foraging activity is found in snakes where the digestion of a single meal may last for several days [59]. But in snakes, one ingestion bout, often a single prey item, fulfills the food requirements for a long period of time [59], [60]. In contrast, their high energy demands plus the poor energy value of their prey forces the sea ducks to repeat the foraging cycle several times in a day in order to ingest the necessary prey biomass. Larger sea duck species seem to forage in a risk-averse strategy where the low energy value of the diet and its poor digestibility are weighted against its low cost and low variance.

A diet based on low quality prey may be beneficial to sea ducks as a competition avoidance strategy. If a species adopts the poorest diet it can afford, it minimizes its number of potential competitors since only species about its own size or bigger can afford to feed on the same array of prey. Bivalves are by far the most widely represented prey in winter diet of sea ducks in North America. However, the mineral shell and low energy value probably precludes smaller sea ducks from feeding on them exclusively. Because of scaling effects smaller species would require a daily prey biomass which would constitute a larger proportion of their body mass than it would for larger species (Fig. 4). But it takes time to find and digest this amount of food. Since sea ducks are mostly diurnal, they are limited by foraging time in winter [8], [19], [20], [21], [42], [43] and it is possible that the small sea duck species could not fit the digestion of such a large amount of shell material within their daily schedule. Therefore, bivalves should not be a reliable food base for smaller sea duck species.

In addition, sea ducks have typically small wings and flight muscles relative to their body mass and high energy cost of flight [38], [45], [46] and the added weight of a gut load could severely impair their flight capacity. Guillemette [44] showed that the amount of mussels taken in a single feeding bout by the common eider may raise its body mass up to a point where the bird can no longer take flight. Flightless sea ducks probably become easy targets for predators like eagles and seals against which escape dives are not appropriate [61], [62], [63], [64], [65], [66]. Therefore, in addition to a possible time constraint which urges the small species to ingest a large amount of food daily, small species are confronted with a contradictory predation risk issue that prompts them to keep their body mass as low as possible. In a much similar respect, Bond and Esler [58] reported that female harlequin ducks exploiting a highly energetic and nearly unlimited food resource did not increase their body mass further than did the females exploiting a much poorer food resource. Based on these observations, these authors proposed that the birds aimed at optimizing body mass rather than maximizing energy intake. Of course, large sea ducks are not exempt from the flight capability issue but in contrast to the small species, the amount of food that meets their needs represents a smaller proportion of their body mass (Fig. 4).

For these reasons, there seems to be a limit to the amount of low quality prey sea ducks can include in their diet. And, since this limit varies positively with body mass, a large individual forager can possibly avoid competition by confining itself to a diet that cannot be afforded by smaller foragers. However, this scaling effect does not completely eliminate the potential for competition and the remaining potential for competition can probably be further avoided with a segregation based on space use and size of prey item. A similar idea of competition avoidance through a scaling mediated diet segregation was put forward by Belovsky [27]. In a comparison of food requirements of herbivores of different body mass this author showed that the amount of shared resources, over which competition may occur, and the amount of exclusive resources in the diet of foragers were largely defined by the scaling of the requirements with body mass. Additional support to this conclusion is found in the study of Goudie and Ankney [25] in Southeastern Newfoundland. These investigators observed that when in sympatry with black scoter and harlequin duck, common eider and long-tailed duck exploited less energetic prey than they did in a site where scoters and harlequin were virtually absent. In our opinion, competition is a latent threat that must be avoided by foraging sea ducks. We believe that avoidance of competition helps to explain the relationship of energy content of the diet with body mass of sea ducks.

The diet composition of larger species was less diversified than that of small species, confirming our second hypothesis stating that the winter diet of larger species is focused on a narrower variety of prey than smaller species. In larger species a single prey category dominates largely in the diet. In contrast, such single prey dominance was less apparent in the diet of smaller ones. It suggests that larger species are more specialized than smaller ones. Also, smaller species fed on a wider array of prey taxa. This was also observed by Goudie and Ankney [48]. This is possibly an effect of the mobile nature of their preferred prey. Unsuccessful foraging dives are not unlikely for prey-quality strategists. On such occasions, taking an alternative and less than optimal prey is surely an appropriate way to secure a reward for the dive effort [48]. The typically long search time inherent in the prey-quality strategy probably prevents these predators from specializing as much as the prey-quantity strategists, who can possibly afford to ignore alternative prey. Another reason is related to the approach of a temporary release in the time constraint. Deteriorating foraging conditions like declining daylight or rising tidal currents [21] probably prompt the foragers in a last intake rush where the digestive constraint becomes less an issue. The foragers should then become less selective and secure as much prey as they can and later proceed with digestion during the subsequent forced fast [67].

Because of the lengthy mechanical crushing of the shell, bivalves remain detectable in the digestive tract for a longer period of time after ingestion than the soft bodied prey that are readily digested [68], [26]. Because of differential digestion rates, our diet composition data are probably skewed toward the bivalves at the expense of soft-bodied prey like the annelids [26]. But in our opinion it is unlikely, given our large sample size, that this bias was sufficiently important to artificially boost the relative importance of bivalves in *Melanitta* and *Somateria* and not in the smaller species. A procedure that helps in checking for the existence of such a bias is to compute the average relative contribution and the frequency of occurrence of each prey taxa and look for discrepancies between the results obtained with both variables. Since in the USBS data set the badly degraded prey items were recorded even if present at trace levels, we were able to do so. A strong correlation between relative contribution and frequency (r_Pearson_ = 0.834; p<0.001) reinforced our confidence in our results. In addition our results are supported by a variety of published data in terms of prey diversity and dominant taxa in the diet of sea ducks (harlequin duck, [55]; long-tailed duck, [69], [70]; *Bucephala*'s, [71]; *Melanitta*'s, [26]; spectacled eider, [49]; common eider, [6]).

The winter diet of sea ducks is probably one of the most energy poor diets ever described. Folivory is another extreme example of low quality diet. It is widely spread among vertebrates but little represented in birds and avian obligate folivory co-occurs with poor flight capacity [72]. Morton [72] highlighted the prohibiting nature of folivory with avian flight requirements, mostly due to low energy reward and heavy gut load associated with this diet. Our results suggest the existence of a lower limit to the quality of an avian diet and the comparatively rare occurrence of avian folivory further supports this idea. In our opinion, the limit is set by the combined effect of weight (mass times gravitational acceleration), physiological, and time constraints.

We propose the following mechanism as possibly shaping the winter diet of sea ducks wintering in the coastal habitats in North America. A metabolic constraint sets a minimum to the amount of food that must be taken on one day, weight and digestive constraints set an upper limit to the size of meals, a time constraint sets a maximum number of meals per day, and competition avoidance causes the ducks to minimize the amount of shared resources in the diet. Our results highlight an ultimate role of body mass in diet composition of predators. But within the boundaries set by body mass, sea ducks probably further define their realized niche in accordance with a variety of other factors. For example, common and Barrow's goldeneyes differ by only a hundred grams of body mass. Their field metabolic rates and food requirements must be accordingly alike. Also, the two goldeneye species are largely sympatric in winter in coastal habitats, at least at large spatial scale [73], [74], [75], giving way to a high potential for competition. But there is a sharp contrast between their diets indicating that they exploit their habitat in completely different ways. The diet segregation we found, regardless of its cause and origin, may reduce competition between sympatric sea ducks.

In conclusion, our study provides clear support to the hypothesis that the energy value of winter diet of sea ducks scales negatively with body mass. We also provide clear evidence of a negative relationship between the diet diversity and body mass. This paper is innovative because:

- it focuses on the role of both animal physiology and inter-specific competition on resource partitioning between species. This has already been done in terrestrial herbivores such as browsers and grazers but rarely in birds;
- it focuses on an entire group of ducks, the Merginae subfamily, where many species face conservation issues and which is poorly known, compared for example to Anatinae;
- it suggests new hypotheses on the evolution of the diet of this group based on metabolic, aerodynamic, digestive and time constraints, as well as on competition avoidance.
